# A phase II randomized trial of individualized neoantigen peptide vaccine combined with unusual radiotherapy (iNATURE) in advanced solid tumors-GCOG0028

**DOI:** 10.3389/fimmu.2025.1538032

**Published:** 2025-08-21

**Authors:** Yan Zhang, Ye-Fan Hu, Lingyu Ma, Yifei Wu, Dandan Chao, Xian Chen, Zhiyuan Xu, Xiaoping Su, Wei Dai, Jiandong Huang, Pingfu Fu, Feng-Ming (Spring) Kong

**Affiliations:** ^1^ Department of Clinical Oncology, University of Hong Kong-Shenzhen Hospital, Shenzhen, China; ^2^ Department of Clinical Oncology, Shenzhen Key Laboratory for Cancer Metastasis and Personalized Therapy, The University of Hong Kong-Shenzhen Hospital, Shenzhen, China; ^3^ The Hong Kong University Shenzhen Hospital Translational Medicine Centre, University of Hong Kong-Shenzhen Hospital, Shenzhen, China; ^4^ School of Biomedical Sciences, Li Ka Shing Faculty of Medicine, University of Hong Kong, Hong Kong, Hong Kong SAR, China; ^5^ Cancer Research Centre, BayVax Biotech Limited, Hong Kong, Hong Kong SAR, China; ^6^ School of Basic Medicine, Wenzhou Medical University, Wenzhou, China; ^7^ Department of Gastrointestinal Surgery, The Second Affiliated Hospital of Wenzhou Medical University, Wenzhou, China; ^8^ Department of Clinical Oncology, Centre of Cancer Medicine, School of Clinical Medicine, Li Ka Shing Faculty of Medicine, The University of Hong Kong, Hong Kong, Hong Kong SAR, China; ^9^ Chinese Academy of Sciences (CAS) Key Laboratory of Quantitative Engineering Biology, Shenzhen Institute of Synthetic Biology, Shenzhen Institutes of Advanced Technology, Chinese Academy of Sciences, Shenzhen, China; ^10^ Guangdong-Hong Kong Joint Laboratory for RNA Medicine, Sun Yat-Sen University, Guangzhou, China; ^11^ Department of Population and Quantitative Health Sciences, Case Western Reserve University, Cleveland, OH, United States; ^12^ Department of Clinical Oncology, Hong Kong University Li Ka Shing Medical School, Hong Kong, Hong Kong SAR, China

**Keywords:** neoantigen vaccine, radiotherapy, immunotherapy, T cell response, low-dose radiation, high-dose radiation, tumor immune microenvironment (TIME), systemic tumor immune environment (STIE)

## Abstract

**Background:**

Neoantigen-based vaccines show promising therapeutic potential in solid tumors such as melanoma, GBM, NSCLC, and CRC. However, clinical responses remain suboptimal in stage IV patients, due to ineffective T-cell function and high tumor burdens. To overcome these limitations, our study investigates a combination strategy using neoantigen peptide vaccines and precision critical lesion radiotherapy (CLERT), which delivers immunomodulatory doses to key tumor regions synergistically enhance immune activation and inhibit progression in multifocal stage IV patients.

**Materials/Methods:**

This is an open-label, multicenter phase II randomized study. The main objective is to evaluate the anti-tumor efficacy of personalized tumor neoantigen peptide vaccines and assess how different radiation doses synergize with vaccination in treating patients with advanced malignant tumors who have progressed after systemic therapy. Patients are stratified by cancer type and randomized 1:1 to receive either placebo with conventional treatment (including high and low dose radiotherapy) or a personalized neoantigen peptide vaccine alongside conventional treatment (including high and low dose radiotherapy). A one-way crossover design is implemented, permitting patients in the placebo arm to transition to the experimental arm upon progression. Clinical outcomes including progression-free survival and objective response rate are assessed both before and after crossover. Key inclusion criteria are as follows: 1) Patients with advanced or recurrent cancers detected by pathology and imaging, who failed first-line treatments; 2) Patients with projected survival ≥3 months and an ECOG score of 0-2; and 3) Patients with at least one predicted high-quality tumor neoantigen.

**Conclusion:**

This trial introduces an innovative combination strategy of precision radiotherapy and neoantigen vaccine. A notable feature of this study is the incorporation of a randomized control and intra-group crossover design, which is rarely utilized in neoantigen trials. The study is designed to provide critical insight into radiation-immune synergy and the clinical benefit of personalized immunization. Additionally, a basket-trial framework is employed, leveraging shared neoantigens across cancer types to improve efficiency and generalizability. This approach may reduce preparation time and cost, facilitating broader implementation of neoantigen-based immunotherapies. Altogether, this trial design represents a significant step toward translational application of tumor neoantigen vaccines and provides a platform for future combinational immunotherapy strategies.

**Trial registration:**

https://clinicaltrials.gov/study/NCT06314087, identifier: NCT06314087; www.chictr.org.cn, identifier: ChiCTR2300078055. Global Collaborative Oncology Group (GCOG) identifier: GCOG0028.

## 1 Introduction

With the advance of tumor immunology, tumor immunotherapy is emerging as “fifth therapeutic pillar” following surgery, radiotherapy, chemotherapy, and targeted therapy (1). Although immune checkpoint blockades (ICBs) targeting PD1 or PD-L1 have been widely utilized across various cancer types, complete responses can only be detected in a fraction of patients, with around 25% overall response rate (2, 3). Especially in advanced-stage patients following conventional first-line and second-line treatments, response rates are relatively low and drug resistance frequently occurred (4). In such cases, personalized and precise treatment approaches, including tumor neoantigens customized for individual patient, are necessary (5). In comparison to traditional tumor-related antigen therapy, this approach offers enhanced specificity, reduced damage to normal tissues, lower risk of autoimmunity, and the ability to target multiple antigens simultaneously (6). This method provides the advantage of activating cytotoxic T cells specifically to eliminate tumor cells (6).

Previous research has demonstrated that neoantigen vaccines can induce neoantigen-specific T cell responses and show promising clinical benefit in certain settings. In 2017, two early-phase clinical studies in advanced melanoma, one led by Ugur Sahin (7) (objective response rate:8/13) and the other by Patrick A. Ott (8) (objective response rate:4/6), reported promising rate of tumor control, particularly in post-surgical patients or those who subsequently received ICBs. Furthermore, neoantigen-based immunotherapies, especially in combination with ICBs, may have potential in other solid tumors, such as gastrointestinal (9), colorectal cancer (10, 11), as well as in a broader range of cancers (12) including non-small cell lung cancer, renal cell carcinoma, urothelial cancer, and triple-negative breast cancer. However, current evidence is limited, as many studies in these cancer types are based on small patient cohorts and lack large-scale clinical validation. For example, a phase 1 trial published in *Nature Medicine* (2025) showed that combining the individualized mRNA vaccine autogene cevumeran with atezolizumab achieved clinical responses in patients unresponsive to immunotherapy (12). In a phase 2 trial conducted by Professor Steve Rosenberg’s team, the combination of neoantigen-reactive tumor-infiltrating lymphocytes (TILs) and pembrolizumab induced responses in 23.5% of patients with refractory gastrointestinal cancers (9). Tumor neoantigen vaccine, or their combination with ICBs, appear particularly promising in patients after surgical resection (8, 13, 14). A phase 1 study in high-risk renal cell carcinoma patients, reported in Nature (2025), demonstrated a 100% recurrence-free survival rate at 40.2 months post-surgery, supporting their use in achieving near-complete clearance of minimal residual disease (15). However, for patients in advanced stages where surgical resection is not an option, the tumor burden is high and it takes time for neoantigen vaccines to elicit an immune response (16). Therefore, combining late-stage therapies controlling local tumor with neoantigen vaccines is considered the optimal treatment approach.

Radiotherapy is the standard treatment for patients with advance cancer, that can eliminate tumor cell by multiple pathways. In addition to direct tumor killing, research indicates that local radiotherapy can trigger systemic responses in the body, particularly post-treatment, leading to increased release of tumor-associated antigens and damage-associated molecular pattern molecules (DAMPs), ultimately inducing immunity or vaccination *in situ (* 17). Additionally, studies have shown that radiotherapy enhances tumor antigen presentation, boosts the variety of tumor-related antigens in draining lymph nodes, elevates T cell recruitment, and enhances the body’s ability to recognize and combat tumors (18, 19). Palma’s phase 2 SABR-COMET study (20) showed that stereotactic ablative radiotherapy (SABR) for metachronous oligometastatic advanced tumors significantly extended survival (41 *vs*. 26 months) but increased adverse reactions, though quality of life remained unaffected. Similarly, Gomez’s trial (21) on non-small cell lung cancer (NSCLC) with synchronous oligometastatic found that local consolidation radiotherapy greatly improved both survival (41.2 *vs*. 17 months) and progression-free survival (14.2 *vs*. 4.4 months). These clinical studies provide strong evidence supporting the effectiveness of local radiotherapy consolidation in advanced lung cancer. Our recent studies further investigated the dynamic changes in various types of immune cells and related factors during radiotherapy treatment. The data revealed that different radiotherapy doses and techniques have varying effects on both the tumor immune microenvironment (TIME) and the circulating immune system (STIE) (22). It is noteworthy that clinical data from large samples across different cancer types indicate that radiotherapy can lead to lymphopenia, which is associated with poor prognosis. Furthermore, alterations in immune factors like Transforming growth factor-beta (TGF-β) (23–25) and Indoleamine 2, 3-dioxygenase (IDO) (26) before and during radiotherapy are closely linked to tumor control and survival, showcasing the diverse patient responses to radiotherapy. Our previous personalized radiotherapy studies have shown that even under the same radiotherapy dose, different patients or tumors exhibit distinct responses to the same treatment regimen (27). For instance, Positron Emission Tomography-Computed Tomography (PET-CT) scans have revealed variations in tumor regression and normal organ damage among patients, illustrating the heterogeneous tumor response to treatment (28). Additionally, we observed that the volume factor, which refers to the changes in tumor volume during treatment, had significant effect on survival, highlighting how treatment response heterogeneity influences the final treatment outcome (29). By utilizing biological metabolic imaging to guide FDG-PET mid-treatment response and employing adaptive radiotherapy technology (BigART) to tailor treatment for stage III NSCLC patients undergoing concurrent chemotherapy, we have demonstrated that optimizing individual doses, enhancing tumor dose delivery, and protecting normal tissues through BigART can ultimately enhance tumor control and prolong survival (30, 31). Multi-center clinical trials have confirmed the aforementioned findings, and we presented the results of RTOG1106 at the 2020 World Lung Cancer Congress (32). Our study achieved the highest ranking in Phase III trials. Recognizing the variability in individual responses to radiotherapy and the potential for toxic side effects, we propose a novel approach known as critical lesion eliminating radiation therapy (CLERT) for stage IV patients. This method follows isotoxicity planning and adheres to radiation dose limits outlined in the NCCN guidelines for organs at risk (OAR), with a dosage limit set at 80% of the recommended level. By implementing precise and personalized radiotherapy, our aim is to optimize therapeutic outcomes while minimizing damage to both the local and systemic immune microenvironments.

Combining radiotherapy and immunotherapy has the potential to enhance both innate and adaptive immunity, ultimately improving patient survival rates. This suggests a potential of synergetic effect with neoantigen vaccines. The immunomodulatory effects of radiotherapy deponed upon various factors such as dose, fractionation, and timing (33). High-dose radiotherapy (HDRT) with doses exceeding 8Gy (33, 34) enhances tumor recognition by T cells, promoting immunogenic cell death (ICD) (34) and T cell activation (35), but also increases regulatory T cells (Tregs) (36) and myeloid-derived suppressor cells (MDSCs) (37–39). HDRT can trigger systemic anti-tumor immunity and synergizes with immune checkpoint blockade (39). Low-dose radiotherapy (LDRT) with doses below 2Gy (33), though less directly tumoricidal, reshapes the tumor microenvironment, suppresses Tregs, MDSCs and rebalances TH1/TH2 (40), and maximizes immunomodulatory effects (activation of dendritic cells (DCs), T cells, NK cells, and B cells) (41) with lower toxicity, enabling multi-site irradiation (41, 42). Various regimens exist in the clinic, with LDRT paired with HDRT, chemotherapy, or immunotherapy for enhanced tumor control (43). Research on radiotherapy combined with neoantigen vaccines remains limited. A preclinical mouse study in 2020 demonstrated that radiotherapy induced the release of neoantigens, which when combined with neoantigen vaccines, led to the expansion of neoantigen-specific CD4+T cells and CD8+T cells, significantly enhancing the anti-tumor effect (18). While these findings are encouraging, it is important to note that results from mouse models do not always translate directly to humans, particularly in advanced cancer stages. Additionally, a clinical trial evaluating the combination of personalized neoantigen vaccines and radiotherapy has been registered in the United States (NCT02287428), although its results have not yet been published. Collectively, these studies suggest (1) Radiotherapy can upregulate the expression of genes containing immunogenic mutations. This means that radiotherapy can increase the visibility of neoantigens to the immune system; (2) Neoantigen-specific CD8+ T cells preferentially kill irradiated tumor cells. This cytotoxic activity relies on the ability of radiation to upregulate class II MHC molecules as well as the death receptors FAS/CD95 and DR5 on the surface of tumor cells. (3) Combining neoantigen vaccines with radiotherapy and chemotherapy might achieve better therapeutic effects. This is because the combination can help overcome immunosuppression in the tumor microenvironment and optimize neoantigen presentation to the immune system. This provides a theoretical foundation for further exploration of neoantigen vaccine combined with radiotherapy.

To date, there is a lack of literature both domestically and internationally regarding the application of personalized radiotherapy in conjunction with tumor neoantigen peptide vaccines for the precise treatment of advanced tumors. Furthermore, our research team has previously conducted studies on tumor tissue gene mutation sequencing analysis, HLA typing sequencing analysis, and the prediction and identification of tumor neoantigens.

This study aims to enroll patients with advanced tumors who have not responded to standard treatments. The study aligns with the international NCCN Clinical Practice Guidelines (2024.v1) and 2023.V3 versions, as well as recent literature by Ionescu et al (44) and Kuang et al (45). It emphasizes the importance of gene sequencing testing as a standard diagnostic method for advanced stage diseases and recommends that current patients be treated in line with these guidelines. The clinical research process of this personalized tumor neoantigen peptide vaccine combined with radiotherapy: (1) Analyzing individual patient tumor tissue sequencing data, tumor tissue RNA transcriptome sequencing, and control blood sample whole exome sequencing data to assess tumor gene mutation, expression levels, and HLA typing; (2) Neoantigen prediction is a critical step in the development of personalized neoantigen vaccines. However, current prediction algorithms differ in terms of their evaluation of MHC binding affinity, peptide processing, and immunogenicity scoring, which can result in variability in predicted candidates. To improve prediction reliability, we utilized three independent teams with complementary algorithms: the TruNeo^®^ platform (46), the ImmuneMirror (47) algorithm, and a Bayesian-based neoantigen prediction model (48). Each pipeline has unique strengths in modeling antigen processing, MHC binding, and immune response likelihood. We integrated their results to prioritize neoantigen peptides that were commonly predicted by at least two algorithms. Immunogenic neoantigens were defined as those with strong binding affinity to patient-specific HLA alleles, confirmed expression in tumor RNA-seq data, and consistent prediction across multiple algorithms, ensuring higher accuracy and minimizing the risk of selecting non-functional peptides”; (3) Synthesizing tumor neoantigen peptide vaccines for *in vitro* and *in vivo* safety testing; (4) Administering precision radiotherapy followed by the individualized tumor neoantigen peptide vaccine clinical trial to evaluate safety, feasibility, and effectiveness. Subjects will provide informed consent before undergoing this combined precision therapy.

## 2 Study design and methods

### 2.1 Study objectives

This study uses patients’ individualized tumor neoantigens to prepare peptide drugs targeting neoantigens, and combines them with precision radiotherapy to study the effectiveness and safety of patients who fail to respond to conventional treatments.

#### 2.1.1 Primary objectives

The primary objective is to evaluate and explore the anti-tumor activity of personalized tumor neoantigen peptide vaccine combined with radiotherapy in the treatment of patients with advanced malignant tumors with the primary endpoint of progression-free survival.

#### 2.1.2 Secondary objectives

The secondary objectives to observe and evaluate the safety of combined peptide vaccines under different radiotherapy doses, and to evaluate the impact of RT dose on the immunogenicity of peptide vaccines on local radiotherapy field response and distant response rate.

#### 2.1.3 Exploratory objectives

The exploratory objectives are to explore the temporal changes in patients’ quality of life and bioactive markers (neoantigen-specific T cells, cytokines, ctDNA and TCR clonotype persistence) before, during and after treatment.

### 2.2 Study design

**Figure 1:**
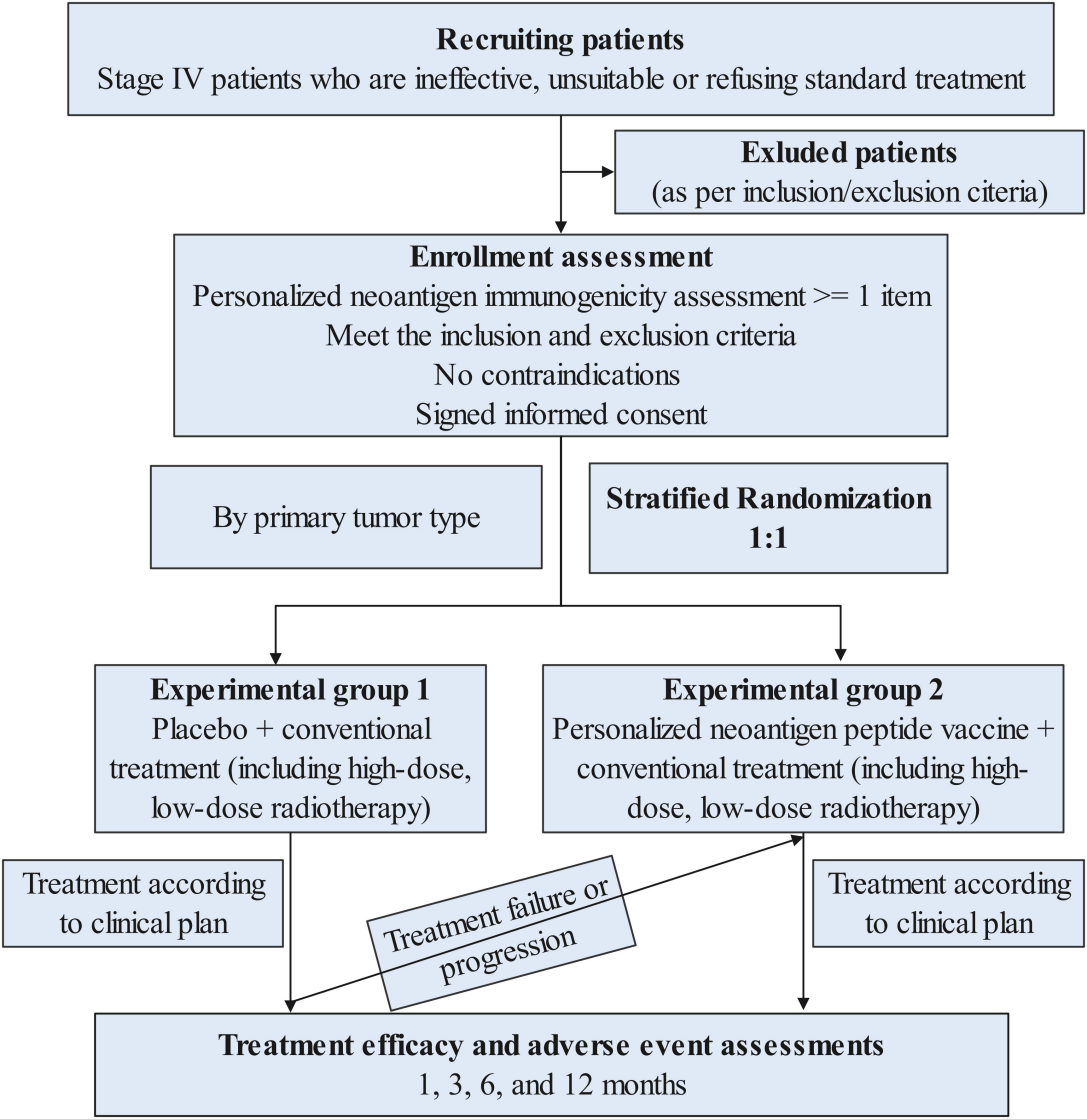
Flowchart of the study design.

The trial is an open-label, multicenter randomized phase II study (**Figure 1**), which includes 2 of the following queues:

**Control group:** placebo + conventional treatment (including radiotherapy) group,

**Intervention group:** personalized tumor peptide vaccine + conventional treatment (including radiotherapy) group,

Subjects will be stratified according to different cancer types and first, second, and third-line treatment stages based on individual condition assessment, and then randomly enter two cohorts in a 1:1 ratio. In addition, there is a one-way crossover experiment (cross-over design) during the research process. Cross-over to the investigational treatment arm (peptide vaccine plus radiotherapy) will be permitted only for patients in the control group who demonstrate radiologically confirmed disease progression, as assessed by RECIST 1.1 and verified by the investigator. Eligibility for cross-over requires that patients meet predefined safety and clinical criteria, including an ECOG performance status of ≤2, absence of Grade ≥3 adverse events, and adequate hematologic, hepatic, and renal function. The proportion, timing, and reasons for cross-over will be systematically recorded and transparently reported. The intervention group will undergo the second phase of personalized tumor peptide vaccine group plus conventional treatment (including radiotherapy), and the response rate of the two phases will be evaluated before and after crossover. In both cohorts, all subjects received radiotherapy, and radiotherapy doses will be further divided into low and high doses. This design will allow us to answer questions about vaccination efficacy and the impact of various radiation doses.

For patients who want to enroll in neoantigen clinical trials, screening for neoantigens may be recommended. It is necessary to take the patient’s fresh tumor tissue (surgery or biopsy or cerebrospinal fluid, pleural effusion, ascites, etc.), and perform whole-exome and whole- transcriptome sequencing on peripheral blood for further clinical development of individualized targeted treatment plans. At the same time, these test data can be used for personalized tumor neoantigen screening in this study. Peptide vaccine therapy requires an 8-week preparation period for the preparation of the peptide vaccine, during which patients continue to maintain standard treatment.

Patients entering the control group’s conventional treatment (including radiotherapy) group will receive conventional treatment plus radiotherapy. Radiotherapy is divided into low-dose and high-dose (first phase); until disease progression occurs, patients can choose to “cross groups” and receive individual treatment. The second stage of treatment consists of tumor peptide vaccine plus radiotherapy (no harm from treatment in the early stage). Patients entering the intervention group ‘s individualized tumor peptide vaccine + conventional treatment (including radiotherapy) group will complete three precise radiotherapy sessions before initiating vaccination with the peptide vaccine. The peptide vaccine treatment will be carried out one week after the end of radiotherapy. The peptide vaccine treatment will last for 5 months. It is a course of treatment until the treatment discontinuation event specified in the protocol occurs. Subjects will continue to undergo post-treatment safety visits, survival follow-ups and tumor progression follow-ups after finishing treatment.

### 2.3 Treatment methods

**Figure 2:**
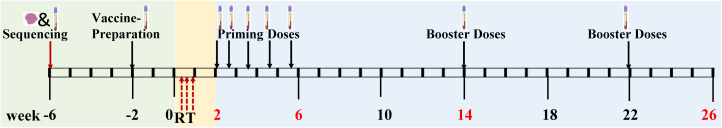
Clinical study treatment regimen.

This study includes personalized neoantigen peptide vaccine treatment and precision radiotherapy. The treatment sequence is radiotherapy first and then vaccine (**Figure 2**). The interval between precision radiotherapy treatment and peptide vaccine is 1 week.

#### 2.3.1 Precision radiotherapy

The course of treatment is 1 week, 3 times in total.

1. Critical lesion eliminating radiation therapy CLERT, in accordance with isotoxicity planning, radiation dose limits are based on standard NCCN (National Comprehensive Cancer Network) guidelines recommended for organs at risk (Organs at risk, OAR) limit dosage to 80% formulation.
2. Critical lesions (Critical lesions) are defined as tumors in critical locations. If the tumor progresses in this location, it will lead to the patient’s death or severe symptoms, affecting the patient’s QOL (the quality of life). Life-threatening tumors such as: central lung cancer; tumors adjacent to large blood vessels, large tracheas, heart, esophagus and other mediastinal tumor lesions; metastatic tumors located in or adjacent to the brainstem. Situations that seriously endanger QOL include, but are not limited to, the following situations: tumors are in the spinal cord, esophagus, portal area, and load-bearing bones, or are close to the rectum, bladder, etc. The final decision was made by a panel of on-study radiation therapy experts.
3. Radiotherapy, SBRT (stereotactic body radiation therapy), the protocol is 8–10 Gy × 3 versus 2 Gy × 3. The radiation dose limit is based on 80% of the OAR limit dose recommended by the standard NCCN guidelines. If the metastases are close to important structures such as the brainstem or esophagus, or have been previously treated with radiotherapy, the radiation dose limit allows the SBRT radiation dose to be adjusted to the OAR guideline limit dose.
4. The key tumor is the treatment target area, which refers to the tumor tissue that mainly affects or threatens the patient’s life or QOL, regardless of the primary site or metastasis, as mentioned above. EDIC (The effective dose to the circulating lymphocyte EDIC, the equivalent dose of circulating lymphocytes) is an important reference indicator for formulating radiotherapy plans. The EDIC is limited to an effective dose that produces grade 2 or higher lymphocyte deficiency in less than 5% of patients. (Patented, part of the research data was disclosed by Jin/Kong, et al. in ASTRO2017).

#### 2.3.2 Polypeptide vaccine treatment

Five-month is a treatment course, and radiotherapy should be started within 1 week after the end of radiotherapy.

1. Receive neoantigen peptide vaccine “ prime phase” treatment on days 1, 4, 8, 15, and 22 of each treatment cycle;
2. “Booster phase” treatment of neoantigen peptide vaccine at weeks 12 and 20;
3. Each vaccination must be carried out after the side effects caused by the previous vaccination have subsided;
4. Dosage and usage: The number of groups of polypeptide vaccine is determined according to the number of neoantigens identified in the patient, and is divided into 2–4 injection pools (1mL per injection). Each pool contains up to 5 peptides, with each peptide dosed at 0.3 mg. Peptides are dissolved in 500 μL of sterile solution and mixed with 500 μL of Poly I:C adjuvant at a concentration of 2 mg/mL prior to subcutaneous injection.
5. Observation: Stay in the hospital for observation until side effects subside.
6. Visit: At the end of each course of treatment, patients must return to the hospital for a visit and start the trial procedure for the next course of treatment.

#### 2.3.3 Neoantigen polypeptide vaccine injection

**Figure 3:**
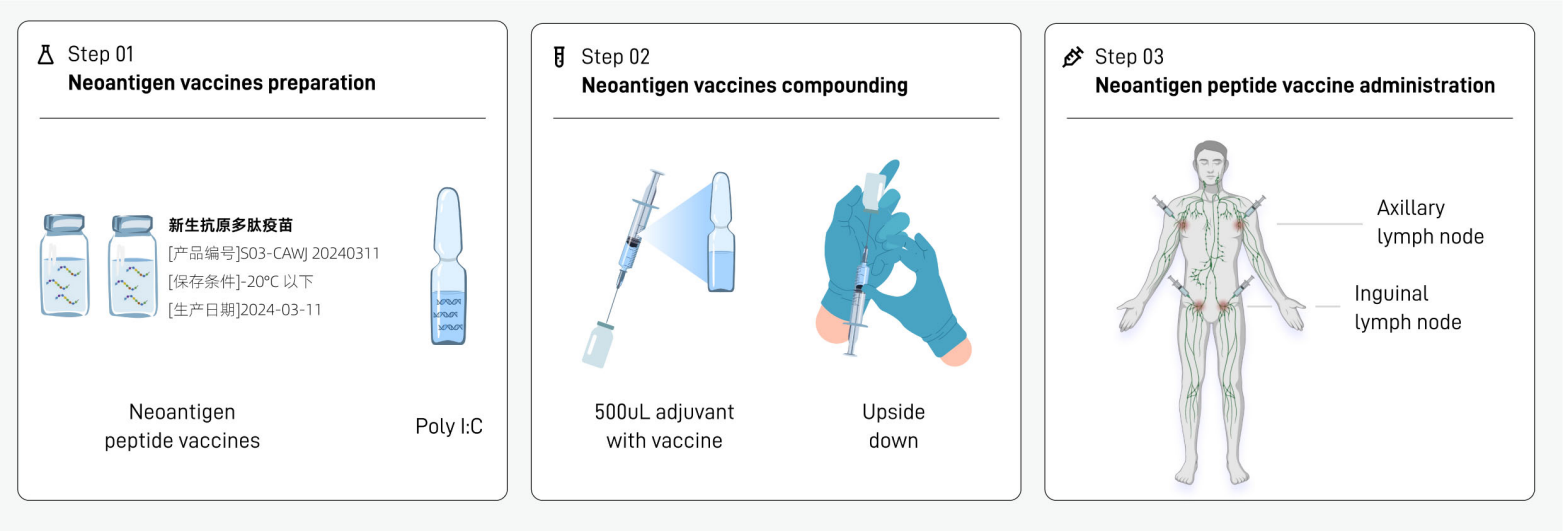
Flow chart of neoantigen peptide vaccine injection.

The preparation, formulation and injection procedures of the neoantigen peptide vaccine are as follows (**Figure 3**):

##### 2.3.3.1 Preparation of neoantigen peptide vaccine (step 01)

1. The drug transporter takes out the whole box of vaccine injection solution for this injection from the transport box, and the nurse confirms that the number on the sealing label of the packaging box is consistent with the injection order.
2. The nurse opens the box and takes out the neoantigen peptide injection (each containing a neoantigen peptide library) and adjuvant poly IC injection (ampoule) in the vial. Poly I:C was used as an immunological adjuvant due to its ability to mimic viral double-stranded RNA, activate dendritic cells via TLR3 and MDA-5 (49), and promote antigen-specific T cell responses.
3. The nurse checks the appearance of each vial:
a. No leaks and cracks
b. The injection solution in the bottle is colorless and has no visible particles.
c. The bottle stopper is not damaged or loose
d. The label number on the bottle is consistent with the bottle number indicated on the injection sheet

##### 2.3.3.2 Mixture of neoantigen peptide vaccine and adjuvant (step 02)

1. The new antigen polypeptide vaccine injection solution can be placed on the table and thawed at room temperature. No special thawing treatment is required and do not shake it violently.
2. Mixing of the new antigen peptide vaccine injection and the adjuvant. The specific operation is as follows:
a. Take a bottle of neoantigen peptide injection (500 uL injection in a vial) and disinfect the bottle stopper with alcohol cotton;
b. Take a bottle of poly I:C injection solution (ampoule), disinfect it with an alcohol cotton pad, break open the bottle cap, and use a 1mL disposable syringe to draw 500uL of poly I:C injection solution.
c. Insert the syringe into the vial of research drug, and inject the poly I:C injection solution in the syringe into the first vial of polypeptide vaccine. After mixing, the mixed solution in the vial is 1 mL.
d. Gently shake the tube upside down to mix the study drug injection and poly I:C injection adjuvant.

##### 2.3.3.3 Injection of neoantigen peptide vaccine (step 03)

1. The injection sites were selected according to the numbering on the injection slip. The four vials of polypeptide vaccine were administered at the left axilla, left groin, right groin, and right axilla, which are lymph node-rich regions commonly used to enhance antigen presentation and immune activation.
2. The requirements for subcutaneous injection are consistent with the conventional vaccination technique, that is, hold the skin taut with the left hand, hold the syringe with the right hand, fix the needle plug with the index finger, and the needle bevel should be upward at an angle of 30 to 40 degrees to the skin. Those who are too thin can pinch the injection site, quickly insert two-thirds of the needle, release the left hand to fix the needle plug, aspirate without blood return, and then inject the liquid medicine.
3. Follow steps 1–2 to complete the injection of the other bottles of new antigen peptide vaccine at the corresponding sites.

### 2.4 Eligibility criteria

#### 2.4.1 Inclusion criteria

1. Voluntarily join this study;
2. Age: 18–80 years old, male or female;
3. Subjects with advanced tumors who have been diagnosed with advanced or recurrent malignant tumors by pathology and imaging, who have received systemic standard treatments that have failed or progressed before enrollment, and who currently have no effective first-line treatments (for effective treatments, please refer to my country’s “Clinical Oncology in China” Recommended by the latest version of the diagnosis and treatment guidelines issued by the “Academic Society”);
4. There is at least one radiologically measurable lesion;
5. Patients with expected survival time ≥ 3 months;
6. Patients with ECOG (Eastern Cooperative Oncology Group) score of 0–2 points;
7. Have had genetic testing, have genome/exome/transcription raw data that meets the basic requirements for neoantigen analysis and prediction, and predict more than one high-quality tumor neoantigen; or have prepared GMP-level neoantigen peptides and *in vitro* immunogenicity evaluation passed;
8. Pregnant women, lactating women, and women of childbearing age who have had a negative pregnancy test within 7 days before enrollment, have no short-term birth plans, and are willing to take protective measures (contraception or other birth control methods) before and during the clinical trial;
9. Good compliance, able to follow the research protocol and follow-up procedures.

##### 2.4.1.1 Neoantigen prediction

1. Sequencing Data Analysis: raw date of the WES and RNA sequencing need to be provided. Prioritize WES data that includes paired tumor tissue and negative control samples sequenced simultaneously.
2. Neoantigen Prediction: neoantigen screening was conducted through three independent predictive analytics platforms, including TruNeo (46), ImmuneMirror (47), and Bayesian (48), and the resulting predictions were collected along with their corresponding neoantigen score rankings. By integrating the results from these three platforms, the top 20 overlapping mutant peptides were selected as targets for the neoantigen vaccine.

#### 2.4.2 Exclusion criteria

1. No neoantigens were found in the sequencing data;
2. Those with a history of bone marrow or stem cell transplantation;
3. Currently participating in other therapeutic clinical trials; clinical trials of traditional Chinese medicine
4. Active bacterial or fungal infection found clinically >= NCI-CTC (Common drug toxicity standards developed by the National Cancer Institute) CTCAE 5.0;
5. Infection with HIV (human immunodeficiency virus), HCV (hepatitis C virus), HBV (hepatitis B virus), severe asthma, autoimmune disease, immune deficiency or patients treated with immunosuppressive drugs;
6. People infected with herpes virus (except for scabs that last for more than 4 weeks);
7. People with respiratory virus infection (except those who have been cured for more than 4 weeks);
8. Severe coronary artery or cerebrovascular disease, or other diseases that the researcher believes should be excluded;
9. Clinical, psychological or social factors affect informed consent or research conduct;
10. Those with a history of drug or peptide allergy, or allergies to other potential immunotherapies;
11. Patients without capacity for civil conduct.

#### 2.4.3 Termination criteria

Subjects have the right to voluntarily withdraw from this clinical study:

In the event of recurrence of the original disease, adverse events, violation of the treatment plan, management or other reasons, the researcher has the right to terminate the clinical study of the subject, and at the same time detail the reasons for terminating the clinical study of the patient in the case report.

Patients will discontinue study treatment prematurely for any of the following reasons:

1. Violation of the protocol: Violation of the main content of the protocol, especially when it is related to the patient’s life safety;
2. Withdraw informed consent;
3. Intolerable adverse reactions, such as allergies or level 4 cytokine release syndrome;
4. Lost to follow-up: unable to be contacted via regular contact methods and beyond the window period;
5. Any other reasons confirmed by the researcher. Patients who withdraw early cannot be replaced by other subjects.

### 2.5 Outcome measures

#### 2.5.1 Primary study endpoints

Progression-free survival (PFS): (Evaluation index: RECIST 1.1, with inclusion of FDG-PET/CT).

#### 2.5.2 Secondary study endpoints

Incidence and severity of adverse events among all treated subjects (evaluation indicators: Treatment toxicity graded according to CTCAE 5, Common Terminology Criteria for Adverse Events 5; overall survival (OS); objective response rate (ORR); In-field response to local radiotherapy; Distant response rate.

#### 2.5.3 Exploratory study endpoints

1. Changes in the immunogenicity of tumor neoantigens before and after treatment, including changes in the immune response of immune cells to tumor neoantigens in peripheral blood before and after treatment. To detect neoantigen-reactive T cells, autologous PBMCs are collected from patients after vaccination and stimulated *in vitro* with their personalized neoantigen peptides. Neoantigen-specific T cell responses are then evaluated using interferon-γ (IFN-γ) enzyme-linked immunospot assay (ELISPOT) experiment to measure the changes in IFN-γ secretion upon neoantigen stimulation;
2. The proportion of immune cells in the peripheral blood of patients before and after treatment. Here, makers including CD3, CD4, CD8, NK1.1 are detected by flow cytometry to screen neoantigen-specific T cells and efficacy markers;
3. Analysis of changes in molecular or genetic indicators related to tumor response or progression before and after treatment. To enhance neoantigen specificity, peptide-MHC tetramers corresponding to predicted neoantigens will be prepared for patients with common HLA types, allowing for *in vitro* enrichment and characterization of neoantigen-specific T cells. scRNA-seq combined with TCR-seq is used to identify neoantigen-specific TCR sequences and analyze the diversification of neoantigen-specific TCR clonotype before and after treatment. If tumor tissue is available, both PBMCs and tumor tissue will be analyzed. In addition, ctDNA and TCR clonotype persistence are monitored, and epitope spreading of vaccine-induced T cell responses is also evaluated by analyzing the emergence or expansion of novel TCR clonotypes at different time points (pre-, mid-, and post-treatment) through TCR sequencing;
4. When biopsy tissue is available, mIHC/IHC analysis can be used to observe changes in the immune microenvironment before and after vaccine treatment; memory T cell responses.

### 2.6 Follow-up

At the end of each course of treatment, patients must return to the hospital for a visit. If necessary and the patient wishes, the trial procedure for the next course of treatment can be started. At the same time, visits must be made every 3 months after each course of treatment. Visits and examinations include blood routine, blood biochemistry, urine routine, stool routine, and coagulation function test on accompanying medications. Carcinoembryonic antigen (CEA) and other tumor markers, CD antigen, T cell activity assessment, vital signs, ECOG score, electrocardiogram, imaging examination (CT, MRI). Please refer to the visit plan for details of the visit plan (**Table 1**). When a certain assessment is required, a “ √ ” will be marked on the visit plan.

**Table 1:** Visit reporting table.

Item	The end of treatment	Post-treatment visit (every 3 months)
Concomitant medication	✓	✓
Blood routine	✓	✓
Blood chemistry	✓	✓
Urine routine	✓	✓
Bowel routine	✓	
Coagulation function test	✓	
Thyroid function test	✓	
Alpha-fetoprotein	✓	
Pregnancy test	✓	
Vital signs	✓	✓
Physical examination	✓	✓
ECOG score	✓	✓
Electrocardiogram	✓	
Echocardiogram	✓	
Blood pressure detection	✓	
Film degree exam	✓	

The follow-up plan lasts for 24 months and is divided into 8 times, that is, every 3 months after the end of treatment, 10 mL of blood will be collected each time for efficacy and safety evaluation, while compliance will be assessed and combined medication status will be recorded.

### 2.7 Security monitoring

#### 2.7.1 Adverse events

Investigators should record all adverse events that occur during drug treatment in a case report form (CRF).

An adverse event refers to any unfavorable change that occurs after taking the study drug or control drug compared with the patient’s baseline (before treatment), including recurrence of other original diseases that occur during the clinical study, regardless of whether its occurrence is related to treatment. Clinically performed invasive examinations themselves are not considered adverse events, but the causes leading to these examinations should be.

Regardless of whether the patient is voluntary or not, all adverse events discovered by investigators, physical examinations, laboratory tests or other methods should be recorded in the CRF “Adverse Event Table”, and treatment and recording should be carefully tracked until recovery. Abnormal laboratory test results alone are generally not considered adverse events unless they are accompanied by clinical symptoms, signs, or require treatment.

When completing the CRF Adverse Event Page, the severity of each adverse event should be appropriately assessed:

1. Duration (start and end dates);
2. Severity: Adverse reactions are classified according to the following criteria according to the most severe degree:
3. Mild: Easier to tolerate, causing only mild discomfort and not affecting daily activities.
4. Moderate: Causes significant discomfort and affects daily activities.
5. Severe: unable to perform daily activities.

Causal relationship to investigational drug:

1. Definitely related: The occurrence of this reaction is consistent with the known reaction type of the suspected drug; it is consistent with the reasonable sequence of events after taking the drug. The adverse reaction is reduced or disappears after the drug is reduced or discontinued, and the same reaction occurs again after the drug is reused.
2. Possibly related: the reaction conforms to the known reaction type of the suspected drug; it conforms to a reasonable time sequence after taking the drug, and the adverse reaction is reduced or disappears after reducing or discontinuing the drug, but the patient’s clinical status cannot reasonably explain the reaction.
3. Possibly related: The reaction is consistent with the known reaction type of the suspected drug; it is consistent with the reasonable time sequence after taking the drug, and the patient’s clinical status or other treatment methods may also produce this reaction.
4. Unlikely: A clinical event, including abnormal laboratory tests, is temporarily related to the time of drug taking, and other drugs, compounds, or the patient’s underlying disease can provide a more reasonable explanation for the event.
5. Unable to evaluate: The information displayed in the adverse event report is insufficient or contradictory, and the information in the report cannot be supplemented or corrected, so it cannot be judged.
6. Irrelevant: It does not conform to the known reaction type of the suspected drug; it does not conform to the reasonable time sequence after taking the drug, and the patient’s clinical status or other reasons can explain the reaction.

The treatment measures and outcomes of adverse events should be recorded on the CRF form. All adverse events should be followed until resolution or stabilization.

#### 2.7.2 Serious adverse events

Serious adverse events include the following:

1. Fatal or life-threatening
2. The patient needs to be admitted to hospital for treatment or extended hospitalization time
3. Resulting in persistent or severe disability/functional impairment
4. The subjects’ offspring have congenital anomalies/birth defects
5. It is medically important or requires intervention to prevent the occurrence of the above-mentioned conditions.

Once the researcher believes that a serious adverse event has occurred, it should be reported to the sponsor within 24 hours after the occurrence and reported to the ethics committee as soon as possible. The case report form for serious adverse events should record the information related to the event as completely and in detail as possible.

Investigators should evaluate and record serious adverse events in the case report form as follows: severity, relationship to the investigational drug, actions taken regarding the investigational drug, and current outcome.

### 2.8 Sample size calculation

This sample size was approximated based on 20% differences between two groups, at 80% detection power.

Calculation method: The primary purpose of this study is to prove that the vaccine treatment group is more effective than the placebo (standard care), with PFS as the main efficacy evaluation index, the planned enrollment time is 24 months, and all subjects are evenly enrolled in the study. The follow-up time is 12 months. According to previous research, it is assumed that the median PFS of the traditional treatment (the control group) is 4 months, and the median PFS of the vaccine group is 10 months (13). The design of crossover of patients who failed in group 1 to group 2 does not affect the statistical results of PFS of the main study. It is based on the PFS before crossover as the statistical calculation.

The 6th month is selected as the interim analysis time node. Considering 0.1 loss to follow-up, 40 patients need to be recruited, with an average of 20 people in each group (**Table 2**). Through the interim analysis, we can make a preliminary validity judgment on the main purpose of this study to determine whether to continue recruiting more patients.

**Table 2:** Sample size calculation for an interim analysis using log rank tests.

Look	Time	–Sample size–		—————Events—————				—Cum.subject time—	
		Group1	Group2	Group1	% of S.S.	Group2	% of S.S.	Group1	Group2
1	6.0	19.2	19.3	6.2	32.1	3.5	18.0	35.6	41.1
2	12.0	38.3	38.5	17.0	44.5	10.4	27.2	98.0	122.8
3	18.0	57.5	57.7	28.7	50.0	18.6	32.2	166.0	217.3
4	24.0	77.0	77.0	40.7	52.9	27.1	35.2	234.9	316.0
5	30.0	77.0	77.0	46.5	60.5	32.3	41.9	268.2	375.1
6	36.0	77.0	77.0	47.7	61.9	33.9	44.0	274.4	393.7

Considering that this study is aimed at pan-tumor and the results of previous studies are based on small samples of liver cancer, we made 4 hypotheticals median PFS prediction values for statistical analysis. The final median PFS was 4 months in the control group and 8 months in the neoantigen vaccine group. The two groups will be enrolled in a 1:1 ratio, α=0.025 (two-sided), β=0.2, and at least 154 subjects with 77 subjects each group are required and the research power reached 80% (**Table 3**).

**Table 3:** Total sample size calculation.

Power	N1	N2	N	Haz ratio (HR)	Ctrl med surv time (M1)	Trt med surv time (M2)	Accrual pat’n	Accrual time/total time	Ctrl loss	Trt loss	Ctrl to trt	Trt to ctrl	Alpha	Beta
0.8028	76	77	153	0.50	4.00	8.00	Equal	24/36	0.10	0.10	0.00	0.00	0.025	0.1972
0.8007	46	47	93	0.40	4.00	10.00	Equal	24/36	0.10	0.10	0.00	0.00	0.025	0.1993
0.8000	481	481	962	0.75	6.00	8.00	Equal	24/36	0.10	0.10	0.00	0.00	0.025	0.2000
0.8003	163	163	326	0.60	6.00	10.00	Equal	24/36	0.10	0.10	0.00	0.00	0.025	0.1997

The sub-group analyses based on high versus low radiation dose are pre-specified exploratory analyses. As there are currently no published studies providing reliable estimates of PFS differences between these sub-groups in the context of neoantigen vaccine therapy, we were unable to perform a dedicated power analysis for the subgroup comparisons. These analyses are intended to be hypothesis-generating and will inform the design of future prospective studies. Additionally, in real-world clinical settings, radiation doses vary considerably among patients, introducing potential confounding effects. To address this, our study design pre-stratified patients into high-dose and low-dose radiation groups rather than allowing radiation prescriptions to vary freely. This approach helps reduce confounding from dose heterogeneity and enhances the interpretability of subgroup findings.

### 2.9 Statistical methods and data analysis

1. The results of this experiment mainly use statistical description methods. The measurement data lists the mean, standard deviation, median, maximum value, and minimum value, and the technical data and grade data list the frequency, rate, and confidence interval.
2. All statistical analysis will be performed using SPSS software.
3. Safety analysis: Mainly descriptive statistical analysis, analyzing adverse events, serious adverse events and adverse reactions of each group of people. Adverse reactions are defined as “definitely related/possibly related, unable to determine” related to this clinical trial.
4. Survival Analysis: Progression-free survival (PFS), the primary endpoint of this study, will be analyzed based solely on data collected prior to cross-over, thereby preserving the integrity and statistical power of the comparison between neoantigen and placebo groups. The Kaplan-Meier method will be used to estimate the median PFS and calculate 95% confidence intervals. Group differences will be compared using the log-rank test. Overall survival (OS), as a secondary endpoint, will be estimated using Kaplan-Meier curves and log-rank tests. To account for potential bias introduced by the cross-over design, we will apply the rank preserving structural failure time (RPSFT) and two-stage methods, as well as inverse probability of censoring weighting (IPCW) to adjust for treatment switching. Additionally, adjusted OS analyses will be conducted by matching patients who cross over with comparable patients in the vaccine group based on key baseline factors (e.g., cancer type, age, performance status), using propensity score matching or multivariable Cox proportional hazards modeling. Sensitivity analyses will include intention-to-treat (ITT), per-protocol, and as-treated populations. Subjects will be stratified according to cancer type and first, second, and third-line treatment stages based on individual condition assessments, ensuring that these factors are appropriately considered in the analysis. Additionally, in the multivariable Cox proportional hazards model for both PFS and OS, cancer type will be included as a covariate, along with other relevant baseline factors, to control for potential confounding effects.
5. Other Effectiveness analysis: Estimate the objective response rate (ORR), disease control rate (DCR) and other efficacy endpoints and improve the 95% confidence interval. The interval estimation uses the Clopper-Pearson method. The effect of lab correlates such as RT dose on clinical outcomes of response and time-to-event will be estimated using logistic regression and Cox mode respectively. The longitudinal data such as quality of life will be estimated using mixed-effect model.
6. Tumor neoantigens response analysis: Neoantigen-specific responses are evaluated by ELISPOT, flow cytometry, and TCR-seq. Peptide–MHC tetramers enrich specific T cells for scRNA/TCR-seq analysis in PBMCs and tumor tissue (if available). TCR clonotype dynamics, ctDNA, and epitope spreading are monitored over time. IHC/mIHC is used to assess immune microenvironment changes when biopsy samples are available.

## 3 Discussion

This clinical trial is the first time that radiotherapy combined with a neoantigen vaccine has been used for phase IV pan-cancer treatment. The response rate of radiotherapy (50, 51) or tumor neoantigen vaccines alone for patients with advanced tumors is only about 30% (52). Although many studies have confirmed that radiotherapy can reshape the immune microenvironment (19), the impact of radiotherapy on tumor neoantigens, the regulation of TIME and the STIE, and the regulatory mechanism of tumor neoantigen vaccines remain unclear. Especially for stage IV patients who are unresectable and have failed multiple lines of treatment, there is an urgent need to conduct clinical trials of radiotherapy combined with tumor neoantigen vaccines to verify the synergistic anti-tumor effect and mechanism. The results will likely provide new treatment opportunities for this population and improve their quality of life.

### 3.1 Radiotherapy dose

Different doses of radiotherapy have distinct immunomodulatory effects. Low-dose radiation can reprogram the TIME to promote immune cell infiltration and activation, which can enhance the effectiveness of immune checkpoint blockade (ICB) (53, 54). On the other hand, high-dose radiation, such as 8 Gy administered in 3 fractions, can increase the expression of immunogenic neoantigens, and is known to induce a stronger ICD response. In 4T1 bearing mouse model, this high-dose radiation regimen showed optimal synergy with a neoantigen vaccine (18). This is likely due to the enhanced release of neoantigens and the subsequent activation of both innate and adaptive immune responses. Similar radiation doses have also been shown to enhance the response to ICB (55). Given the importance of radiotherapy dose on TIME, this clinical trial also randomly divided each group into two subgroups: high-dose and low-dose, to explore the immunomodulatory mechanism of radiotherapy dose. We hypothesize that the high-dose regimen will result in stronger ICD, particularly by promoting a more robust release of neoantigens, thereby enhancing the efficacy of neoantigen vaccination. In contrast, while the low-dose regimen may have a more limited direct impact on tumor control, it could still contribute to immune activation, especially when combined with the vaccine.

### 3.2 Clinical trial design

Published clinical trials of neoantigen vaccines have predominantly been single-arm studies, limiting the ability to thoroughly analyze the efficacy of the vaccine (6). This clinical trial represents a novel approach with a two-arm, randomized group design, allowing for a more comprehensive evaluation of the effectiveness of neoantigen vaccines without inherent bias. In comparison to single-arm studies, this design offers a more robust evidence-based foundation for the clinical implementation of neoantigen vaccines.

This trial design was informed by previous neoantigen vaccine studies, particularly Ott, et al. (8), *Nature* 2017. While the neoantigen peptide design concept is similar, our study introduces several important differences: we employ a randomized controlled design rather than a single-arm study; incorporate radiotherapy (both low- and high-dose) rather than vaccine monotherapy; and include a one-way crossover arm to further evaluate vaccine efficacy. These improvements are aimed at increasing clinical efficacy and evaluating the synergistic potential of radiotherapy and neoantigen vaccines.

To facilitate the inclusion of patients in the control group for neoantigen vaccine treatment, a crossover program has been implemented. This program enables subjects in the control group with ineffective treatment progress to transition to the vaccine treatment group. Such a clinical trial design not only addresses the need for unbiased efficacy evaluation but also creates new treatment opportunities for a broader patient population. Also, cross-over design needs fewer participants compared to a standard parallel randomized controlled trial (RCT).

### 3.3 Shared neoantigens

This study was designed to mimic a basket experiment for patients with pan-cancer shared neoantigens harboring the same gene mutation, such as TP53, KRAS, EGFR. A universal shared neoantigen vaccine could be developed, allowing any patient with a tumor containing a specific genetic mutation to be eligible for treatment with a targeted neoantigen vaccine (56). This approach addresses rare tumor populations with shared neoantigens, particularly benefiting rare tumor patients lacking new treatment options and facing challenges in conducting independent clinical trials. Furthermore, the study encompasses pan-cancer species, facilitating the identification of shared neoantigens across different cancer types and evaluating the broad applicability of neoantigen vaccines. Consequently, if a patient matches the known shared neoantigen and HLA typing, the neoantigen-specific T cell response can be confirmed *in vitro* using pre-prepared shared neoantigen peptides. In case of an immune response, the patient can promptly receive neoantigen therapy, leading to significant reductions in both time and costs associated with personalized neoantigen screening.

We actively explore the presence of “shared” neoantigens during the patient screening and selection process in our trial. Based on the current cohort of screened patients, we have identified some recurrent neoantigens shared across individuals, such as mutations in KRAS and EGFR. These shared neoantigens may offer potential as broader immunogenic targets and will be further investigated in our ongoing analysis.

This clinical trial is a randomized, two-arm study investigating the combination of radiotherapy with a neoantigen vaccine in patients who have experienced advanced treatment failure. The primary aim is to validate the efficacy of this combined treatment approach, while also examining the immunomodulatory effects and potential synergistic anti-tumor mechanisms. The trial commenced patient recruitment in January 2024 and aims to enroll 154 advanced pan-cancer patients over the next 2–3 years.
